# Pure FPGA Implementation of an HOG Based Real-Time Pedestrian Detection System

**DOI:** 10.3390/s18041174

**Published:** 2018-04-12

**Authors:** Jian Hua Luo, Chang Hong Lin

**Affiliations:** Department of Electronic and Computer Engineering, National Taiwan University of Science and Technology, #43, Sec. 4, Keelung Rd., Taipei 106, Taiwan; chlin@mail.ntust.edu.tw

**Keywords:** HOG, SVM, FPGA, hardware acceleration, pedestrian detection

## Abstract

In this study, we propose a real-time pedestrian detection system using a FPGA with a digital image sensor. Comparing with some prior works, the proposed implementation realizes both the histogram of oriented gradients (HOG) and the trained support vector machine (SVM) classification on a FPGA. Moreover, the implementation does not use any external memory or processors to assist the implementation. Although the implementation implements both the HOG algorithm and the SVM classification in hardware without using any external memory modules and processors, the proposed implementation’s resource utilization of the FPGA is lower than most of the prior art. The main reasons resulting in the lower resource usage are: (1) simplification in the Getting Bin sub-module; (2) distributed writing and two shift registers in the Cell Histogram Generation sub-module; (3) reuse of each sum of the cell histogram in the Block Histogram Normalization sub-module; and (4) regarding a window of the SVM classification as 105 blocks of the SVM classification. Moreover, compared to Dalal and Triggs’s pure software HOG implementation, the proposed implementation‘s average detection rate is just about 4.05% less, but can achieve a much higher frame rate.

## 1. Introduction

Real-time pedestrian detection is an important technology for modern society [1] in many applications, such as surveillance [2], intelligence vehicle systems [3], and robot navigation [4]. Some studies have extracted diverse features from an image and have found appropriate classification methods to perform robust pedestrian detection [5,6,7,8,9]. An excellent algorithm, the histogram of oriented gradients (HOG) [10], was proposed by Dalal and Triggs in 2005. It is an efficient feature extraction algorithm, and it can accurately detect a pedestrian in difficult conditions, such as deformation, rotation, or illumination changes. However, the calculation cost of the HOG is very high because of its repeated and complex computation. However, several studies have realized pedestrian detection based on a central processing unit (CPU) or graphics processing units (GPUs), or the combination of both [11,12,13,14,15], they are not suitable to many applications, such as surveillance. Since such applications are contingent on hardware cost and power consumption, field programmable gate arrays (FPGA), with better speed and power consumption, are more suitable than GPUs.

Some prior works have realized pedestrian detection based on a FPGA, such as [16,17,18,19,20,21,22]. In early years, Kadota et al. [16] proposed several methods to simplify the computation, such as the square root and arctangent. Negi et al. [17] proposed an implementation by using binary-patterned HOG features, adaptive boosting (AdaBoost) classifiers [23], and some approximation arithmetic strategies. Hsiao et al. [18] realized an implementation in an embedded hardware/software co-design. Komorkiewicz et al. [19] proposed an accurate system using single-precision 32-bit floating point computations in all stages of image processing. Hiromoto et al. [20] proposed a window-based scanning architecture. Mizuno et al. [21] proposed a simplified HOG algorithm with cell-based scanning and utilized an external SRAM to assist support vector machine (SVM) calculation [24]. Hahnle et al. [22] proposed a cell-based scanning method, as well.

The above implementations have at least one characteristic from the following list: (a) they implement the HOG without implementing any classification; (b) they use external memory or an external processor in their implementation; and (c) they use large hardware resources in their implementation.

To avoid using additional resources, the proposed implementation is all in a FPGA with a digital image sensor, which is responsible for capturing images and it does not use any external memory modules and processors. Moreover, the implementation can be suited to certain environments, such as surveillance, under reasonable resolution and resource utilization.

Shown in the experimental results, although the proposed implementation implements both the HOG algorithm and the SVM classification in hardware without using any external memory modules and processors, the implementation’s resource utilization of the FPGA is lower than most of the prior art. The main reasons resulting in the lower resource utilization are shown as follows:

1. Through simplifications in the Getting Bin, the proposed system can use fewer hardware resources to obtain bins.
2. Through the distributed writing and two shift registers in the Cell Histogram Generation sub-module, the proposed system can easily deal with each intermediate cell histogram without any address decoder.
3. To decrease the number of calculations, the proposed system reuses each sum of the cell histogram, which is overlapped between each block in the Block Histogram Normalization sub-module.
4. The same as some previous state of the art, we regard a window of the SVM classification as 105 blocks of the SVM classification.

The detail of this article is organized as follows: The background knowledge of the HOG feature extraction algorithm and SVM classification are introduced in Section 2. Section 3 explains how the pedestrian detection is implemented using the HOG algorithm and the SVM classification on a FPGA. Subsequently, the implementation’s hardware resource utilization, detection rates, and comparisons with previous works are shown in Section 4. Finally, the article concludes in Section 5.

## 2. Background Knowledge

**Figure 1:**
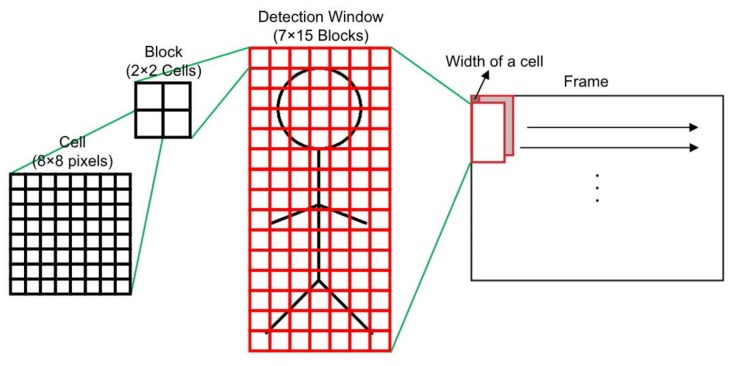
Division of a detection window in a frame in the proposed scheme.

This study implements not only the HOG feature extraction algorithm, but also the SVM classification on a single FPGA. The pedestrian detection in each frame is performed using a sliding window. As shown in Figure 1, the detection window in the proposed scheme consists of 64 × 128 pixels in size, and it contains 7 × 15 blocks. A block further contains 2 × 2 cells and a cell contains 8 × 8 pixels. It slides the detection window rightward or downward by eight pixels (the width of a cell) per time in a frame.

The pedestrian detection process consists of two parts: First, it obtains the descriptors of each detection window by using the HOG feature extraction algorithm. Afterwards, the descriptors are classified by using the SVM classification.

### 2.1. HOG Feature Extraction Algorithm

The process of the HOG feature extraction consists of three steps:

1. Calculation of Gradients

Before calculating the gradients, the proposed system converts *RGB* values of pixels to gray values of pixels. In this scheme, the converted method is realized by the Equation (1) [25]:

(1) Gray=R×0.299+G×0.587+B×0.144

Let *Gray*(*i*, *j*) be the gray values of the pixel (*i*, *j*). The horizontal differences of gray *Gx*(*i*, *j*) and vertical differences of gray *Gy*(*i*, *j*) are defined as shown in Equations (2) and (3):

(2) Gx(i, j)=Gray(i+1, j)−Gray(i−1, j)

(3) Gy(i, j)=Gray(i, j+1)−Gray(i, j−1)

Using these values, gradient magnitudes *M*(*i*, *j*) and gradient orientations *θ*(*i*, *j*) are calculated according to Equations (4) and (5), respectively:

(4) $$M\left(i,\;j\right)=\sqrt{{\mathrm{Gx}(i,\;j)}^{2}+{\mathrm{Gy}(i,\;j)}^{2}}$$

(5) $$\theta \left(i,\;j\right)=\tan^{-1}\left(\frac{\mathrm{Gy}(i,\;j)}{\mathrm{Gx}(i,\;j)}\right)$$

2. Cell Histogram Generation

When magnitudes *M*(*i*, *j*) and orientations *θ*(*i*, *j*) are obtained, they are utilized to vote for generating cell histograms. In this scheme, orientations are divided into nine bins. In each cell, gradient magnitudes of all pixels are voted on their respective bins. The contributions of all pixels in a cell are added up to create a histogram.

3. Block Normalization

In this scheme, each group of 2 × 2 cells, which is regarded as a block, is normalized by using the L1-sqrt normalization method:

(6) $$v\to \sqrt{\frac{v}{\left({\left\|v\right\|}_{1}+e\right)}}$$

where v is an unnormalized descriptor vector in a block, ${\left\|v\right\|}_{1}$ is its 1-norm, and e is a small constant. Finally, all of normalized blocks are concatenated as HOG descriptors ***x***.

### 2.2. SVM Classification

When obtaining HOG descriptors, they are classified by the linear SVM classification equation:

(7) $$y\left(x\right)=\omega^{T}\cdot x+b$$

In the training section of this study, firstly, every 64 × 128 pixels of an image would be converted into 3780 HOG features through the HOG feature extraction algorithm. Two classes are used in the HOG: with a pedestrian and without a pedestrian. Subsequently, the library of the SVM supported in OpenCV would use these HOG features to train the weight vector ω and the bias *b*. When the weight vector ω and the bias *b* are determined after the training section, they would be stored into the ROM on the FPGA, and then the implementation on the FPGA utilizes it to classify new 64 × 128 pixels of the detection windows.

## 3. Proposed Hardware Implementation

This section explains how the proposed system implements pedestrian detection using the HOG algorithm and the SVM classification in real-time on a FPGA. From the inputs of the pixels to the outputs of the results, the entire implementation is accomplished entirely in hardware.

### 3.1. Flow of Pedestrian Detection Algorithm

**Figure 2:**
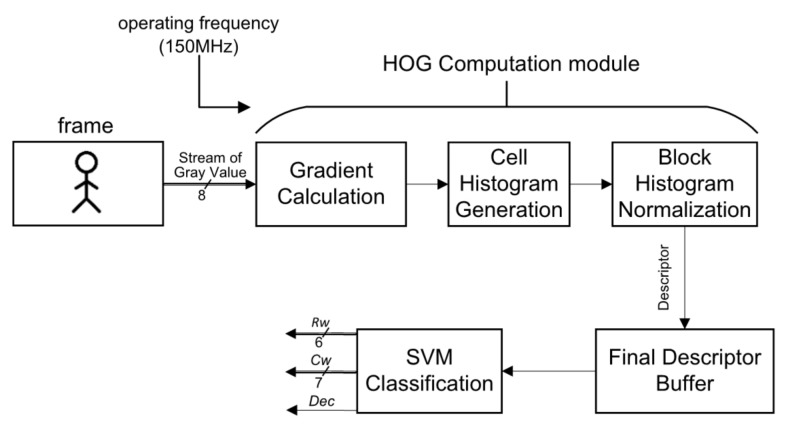
Flow of the proposed system’s pedestrian detection algorithm.

As shown in Figure 2, each pixel (*i*, *j*) in the input frame (resolution of 800 × 600), which is captured by the digital image sensor is converted to gray values, *Gray*(*i*, *j*) by Equation (1). The key performance parameters of the digital image sensor are listed in Table 1.

To avoid expensive floating point computations, the *RGB* weights are shifted by eight bits (0.299 << 8 ≈ 77, 0.587 << 8 ≈ 150, 0.144 << 8 ≈ 29) to retain their decimal information. The HOG Computation module, which contains three sub-modules and the SVM Classification module are run at a 150 MHz operating frequency. The *Gray*(*i*, *j*) are continually streamed into the Gradient Calculation sub-module and then their gradients are determined. When their magnitude and their orientation are produced, the cell histogram is generated in the Cell Histogram Generation sub-module. Finally, in the Block Histogram Normalization sub-module, a group of cell histograms (2 × 2 cells comprise a block) is normalized as the final HOG features for the SVM classification. After classification, three signals are outputted:

- The row of the detection window position, *Rw*.
- The column of the detection window position, *Cw*.
- The result of the detection window (1 with pedestrian, 0 without pedestrian), *Dec*.

In the following sections each sub-module and the SVM Classification module in Figure 2 are individually described.

### 3.2. Gradient Calculation

**Figure 3:**
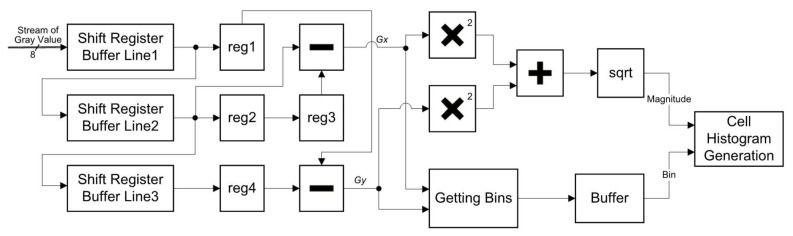
Diagram of the Gradient Calculation sub-module in the proposed structure.

Figure 3 is the implementation of the gradient calculation sub-module in the proposed structure. The *Gray*(*i*, *j*) is continually streamed into a shift register, which is composed of three line buffers. Each line buffer consists of 800 8-bit words to suit the system’s column size of a frame. When the shift register is appropriate for computing differences, data is buffered to calculate horizontal differences *Gx*(*i*, *j*) and vertical differences *Gy*(*i*, *j*). When *Gx*(*i*, *j*) and *Gy*(*i*, *j*) are obtained, magnitudes *M*(*i*, *j*) and orientations *θ*(*i*, *j*) are calculated simultaneously. The result of sqrt in Figure 3 is obtained by the Altera’s ALTSQRT IP core [26]. The Getting Bins (computing orientations) is described in Figure 3. Results of the Getting Bins are buffered because the results of the Getting Bins are faster than the results of the computing magnitudes.

**Figure 4:**
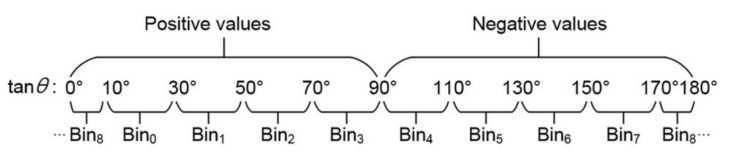
Dividing orientations into nine bins in the proposed scheme.

The original HOG divided the orientations of gradients into several bins, and used the magnitudes of each gradient as the weight to generate the cell histogram in the Cell Histogram Generation sub-module. Then, the distribution of the cell histogram is computed in the Block Histogram Normalization sub-module to obtain the final feature. In the proposed scheme, it divides orientations into nine bins (as shown in Figure 4).

To avoid the expensive floating point computation of arctangents, this study refers to the scheme of Kadota et al. [16] and Zhou et al. [27], which uses a compared method to obtain corresponding bins. Firstly, the study utilizes Algorithm 1 to determine *θ*(*i*, *j*)’s rough range.

**Algorithm 1.** Determine *θ*(*i*, *j*)’s rough range.Input:   *Gx*(*i*, *j*), *Gy*(*i*, *j*)Output:*Gx_r*(*i*, *j*): absolute values of *Gx*(*i*, *j*) *Gy_r*(*i*, *j*): absolute values of *Gy*(*i*, *j*) *GxGy_xor*(*i*, *j*): values determining *Gx*(*i*, *j*) and *Gy*(*i*, *j*) have same sign or not① if (*Gy*(*i*, *j*) == 0) begin //*θ(i*, *j*) = 0°, *bin* (*i*, *j*) = 8   *GxGy_xor*(*i*, *j*) = 0;   *Gx_r*(*i*, *j*) = 10;   *Gy_r*(*i*, *j*) = 1 << 8; ② end else if (*Gx*(*i*, *j*) == 0) begin //*θ(i*, *j*) = 90°, bin (*i*, *j*) = 4   *GxGy_xor*(*i*, *j*) = 1;   *Gx_r*(*i*, *j*) = 1;   *Gy_r*(*i*, *j*) = 3 << 8; ③ end else begin //0° < *θ(i*, *j*) < 90° or 90° < *θ(i*, *j*) < 180°, *bin* (*i*, *j)* hasn’t been determined   *GxGy_xor*(*i*, *j*) = *Gx*[bit of sign] (*i*, *j*) ^ *Gy*[bit of sign] (*i*, *j*);   *Gx_r*(*i*, *j*) = *Gx*[bit of sign] (*i*, *j*) ? (~*Gx*[bit of sign-1:0] (*i*, *j*)) + 1 : *Gx*[bit of sign-1:0] (*i*, *j*);   *Gy_r*(*i*, *j*) = *Gy*[bit of sign] (*i*, *j*) ? ((~*Gy*[bit of sign-1:0] (*i*, *j*)) + 1) << 8: (*Gy*[bit of sign-1:0]   (*i*, *j*)) << 8;  end

Afterwards, the nearest tangent value of *Gy*(*i*, *j*) is determined in Algorithm 2. Finally, the exact *bin* (*i*, *j*) is obtained in Algorithm 3.

**Algorithm 2.** Determine the nearest tangent value of *Gy_r*(*i*, *j*).  Input:  *Gx_r*(*i*, *j*), *Gy_r*(*i*, *j*)  Output:*Distance_sign_k_* (*i*, *j*): indicate *Gx_r*(*i*, *j*) × tan*θ*(*i*, *j*) < *Gy_r*(*i*, *j*) or *Gx_r*(*i*, *j*) × tan*θ*(*i*, *j*) ≥ *Gy_r*(*i*, *j*) *NearestDeg* (*i*, *j*): flag of the nearest degree① //fixed tan*θ* are shifted by 8 bits to retain their decimal information. *Distance_0_* (*i*, *j*) = *Gx_r*(*i*, *j*) × (tan10° << 8)—*Gy_r*(*i*, *j*) *Distance_1_* (*i*, *j*) = *Gx_r*(*i*, *j*) × (tan30° << 8)—*Gy_r*(*i*, *j*) *Distance_2_* (*i*, *j*) = *Gx_r*(*i*, *j*) × (tan50° << 8)—*Gy_r*(*i*, *j*) *Distance_3_* (*i*, *j*) = *Gx_r*(*i*, *j*) × (tan70° << 8)—*Gy_r*(*i*, *j*) ② ${\mathrm{Distance}\_\mathrm{abs}}_{k}\;\left(i,\;j\right)=\{\begin{array}{c} {\mathrm{Distance}}_{k}\;\left(i,\;j\right)<0,\;{\mathrm{Distance}}_{k}\;\left(i,\;j\right)\;\mathrm{takes}\;2^{\prime }s\;\mathrm{complement}\\ {\mathrm{otherwise},\;\mathrm{Distance}}_{k}\;\left(i,\;j\right) \end{array}$ ${\mathrm{Distance}\_\mathrm{sign}}_{k}\;\left(i,\;j\right)={\mathrm{Distance}}_{k}[\mathrm{bit}\;\mathrm{of}\;\mathrm{sign}]\;\left(i,\;j\right)$, 0≤k≤3 ③ $\mathrm{NearestDeg}\;\left(i,\;j\right)=\mathrm{minDegree}\left({\mathrm{Distance}\_\mathrm{abs}}_{k}\;\left(i,\;j\right),\;0\leq k\leq 3\right),\;\mathrm{minDegree}()=k$

Through proposed Algorithms 1–3, the proposed system only uses 220 LUTs, 235 registers, and four DSP blocks to implement the comparison method to get bins. The four DSP blocks are mainly used to implement the step ① in Algorithm 2.

**Algorithm 3.** Obtain *bin* (*i*, *j*) according to the property of tan*θ*.  Input:  *GxGy_xor*(*i*, *j*), *NearestDeg* (*i*, *j*), *Distance_sign_k_* (*i*, *j*)  Output:*bin* (*i*, *j*)① if (*GxGy_xor*(*i*, *j*) == 0) //Same sign  case(*NearestDeg* (*i*, *j*))   0: *θ(i*, *j*) = 10°   1: *θ(i*, *j*) = 30°   2: *θ(i*, *j*) = 50°   3: *θ(i*, *j*) = 70°  end case  if *Distance_sign_k_* (*i*, *j*) == 1 (|*Gx*(*i*, *j*)| × |tan*θ(i*, *j*)| < |*Gy*(*i*, *j*)|)   the *bin* (*i*, *j*) is at big degree side of the nearest *θ(i*, *j*)  Else   the *bin* (*i*, *j*) is at small degree side of the nearest *θ(i*, *j*) ② else //*GxGy_xor*(*i*, *j*) == 1 (Different sign):  case(*NearestDeg* (*i*, *j*))   0: *θ(i*, *j*) = 170°   1: *θ(i*, *j*) = 150°   2: *θ(i*, *j*) = 130°   3: *θ(i*, *j*) = 110°  end case  if *Distance_sign_k_* (*i*, *j*) == 1   the *bin* (*i*, *j*) is at small degree side of the nearest *θ(i*, *j*)  Else   the *bin* (*i*, *j*) is at big degree side of the nearest *θ(i*, *j*)

### 3.3. Cell Histogram Generation

**Figure 5:**
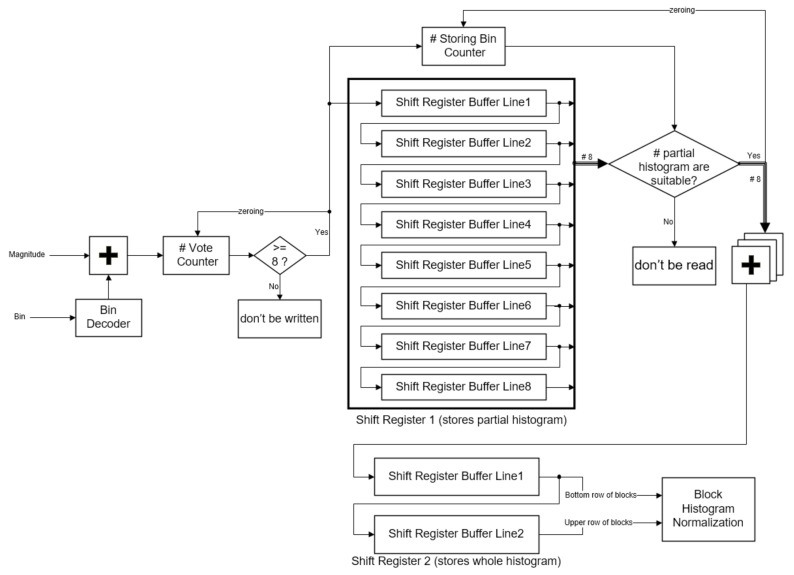
Diagram of the Cell Histogram Generation sub-module in the proposed structure.

When the magnitudes *M*(*i*, *j*) and *bin* (*i*, *j*) are obtained, they are utilized to vote for generating cell histograms (as shown in Figure 5). Each *M*(*i*, *j*), which is regarded as a weight, refers to *bin* (*i*, *j*) to vote, directly. In the proposed scheme, the results of voting magnitude for every pixel are not written into Shift Register 1 until the results of voting magnitudes for eight pixels (width of a cell) are completed.

Shift Register 1 contains eight buffer lines and each buffer line stores 100 partial cell histograms. Because a frame in the scheme contains 100 columns of cells and every cell contains nine bins, a buffer line contains 900 12-bit words, and every word stores a partial value of a certain bin. Storing Bin Counter is able to compute how many partial values have been stored into Shift Register 1. When the amount of the stored partial values are suitable for summing, every partial value in the same bin would be read out and summed to the whole cell histograms. The whole cell histograms would be stored into Shift Register 2. When 100 columns by one row of whole cell histograms are obtained, the next 100 columns by eight rows of partial cell histograms would be computed in the same way.

Shift Register 2 contains two buffer lines, because the shift register could be suited to block overlapping. Each buffer line in the shift register contains 900 13-bit words to store the histogram of the whole cell. Since every block overlaps adjacent blocks in a cell, this study utilizes the two buffer lines of the shift register to easily assemble the blocks. When the shift register is appropriate for the next phase, the data in the shift register would be read by the Block Histogram Normalization sub-module.

Through the proposed distributed writing and two shift registers, it can easily deal with each intermediate cell histogram without any address decoder.

### 3.4. Block Histogram Normalization

**Figure 6:**
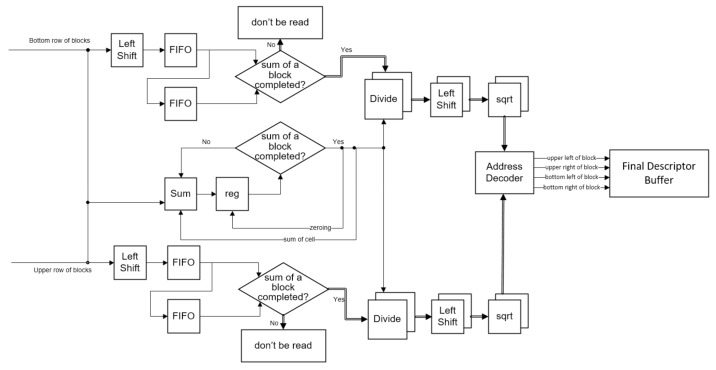
Diagram the Block Histogram Normalization sub-module in the proposed structure.

In the Block Histogram Normalization sub-module (as shown in Figure 6), this study uses the L1-sqrt normalization method to realize the normalization. Firstly, the cell histograms read from the shift register are inputted to Left Shift and Sum. Normally, results of the block histogram normalization are less than 1, so Left Shift shifts the data for saving their decimal information. Through these left shifts in Figure 6, results of Divide and sqrt (both from Altera’s IP core [26]) would not be zeroes. Since Divide would not be run until the cell histograms are added as a sum of the blocks, the proposed system inputs these data into FIFOs. When the cell histograms are added as a sum of the blocks, the data would be read from FIFOs into Divide and then the data would be divided by the sum of the blocks. To reduce the computational workload, this study exploits the property of block overlapping. Every block overlaps adjacent blocks by the width of a cell. When the overlapping cell is read into Divide, it would be also write into another FIFO for the next block histogram computation. For the same reason, a sum of the cells would be stored and it would be added for the next sum of the blocks. Finally, the results of Divide are shifted and inputted into the sqrt to produce the final HOG descriptors. Through an address decoder, four descriptors, which individually indicate a cell of a block (a block has four cells), would be buffered to the Final Descriptor Buffer.

### 3.5. SVM Classification

This section explains the SVM Classification module, which was implemented on the FPGA as well. The linear SVM classification (Equation (7)) is implemented in this module. The weight vectors ω and the bias *b* are stored in a ROM on the FPGA. The detection window in the proposed scheme contains 7 × 15 blocks and every block contains 36 descriptors, so a detection window contains 7 × 15 × 36 = 3780 descriptors. If it waits for all of the descriptors to consist of a whole window (3780 descriptors) to begin the SVM classification, it has to use a large amount of memory to store the previous descriptors. To reduce the memory utilization, the same as some previous states of the art, the SVM Classification module in this study uses a cell-based scanning structure. It modifies the linear SVM classification Equation (7) as Equation (8), in which the $x_{\mathrm{Bi}}$ means descriptors of a block in a window:

(8) $$y\left(x\right)=\sum_{i=0}^{105}(\omega_{i}^{T}\cdot x_{\mathrm{Bi}})+b$$

Since a window contains 7 × 15 = blocks, a window of the SVM classification can be regarded as 105 blocks of the SVM classification. Hence, when the descriptors of a block are completed, we can begin these partial SVM classifications. In this case, rather than store descriptors of many windows, it just stores the results of partial SVM classifications.

**Figure 7:**
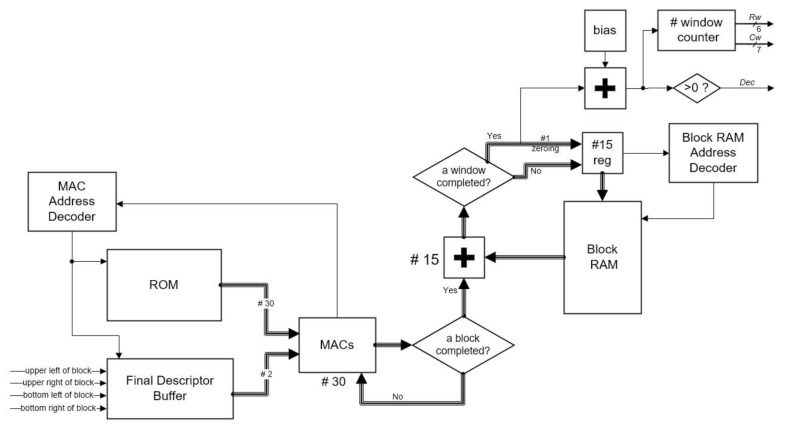
Diagram of the SVM classification in the proposed structure.

Since a window contains 15 rows of blocks, the Block RAM in Figure 7 consists of 15 rows of RAM. Each row of RAM consists of a row of 93 windows in which each word stores their results of partial SVM classifications individually. The proposed system uses two multiply-accumulators (MACs) for a row of RAM, so it has 30 MACs in the SVM Classification module. Since a block may overlap 105 windows, at most, descriptors of a block may have to take the dot product of the whole weight vectors in ROM. Hence, two descriptors read from the Final Descriptor Buffer are inputted to each MAC, and 15 weight vectors read from ROM are used for each descriptor (30 vectors in total). A MAC Address Decoder is responsible for reading/writing the Final Descriptor Buffer and determines which weight vectors should the current descriptors to be taken dot product to. When a block is completed, these 15 results of partial SVM classifications are added to their previous results of partial SVM classifications stored in Block RAM and then the results are judged whether a window is completed. If a window is completed, the completed result is added to judge whether the window contains any pedestrians, the according word in the RAM would be zeroed, and another 14 results would be rewritten according to the words. If a window is not completed, 15 results would all be rewritten according to the words. After the words in a row of RAM are zeroed, the next row of partial results would be stored to these zeroed words. Hence, the 15 rows of RAM can be reused for all windows.

Since the blocks (except the first one) in a frame need 126 cycles to be completed for partial SVM classifications, a frame of blocks would be completed in 126 × 99 × 74 (a frame contains 99 × 74 blocks) = 923,076 cycles. If the operating frequency of the proposed hardware is 150 MHz, it can achieve around 162 fps.

## 4. Results and Discussion

The proposed system is implemented on an Altera tPad FPGA board, developed by Terasic (Hsinchu City, Taiwan), which has a DE2-115 evaluation board with a Cyclone IV EP4CE115 FPGA. The entire implementation is accomplished all in hardware without using any external memory modules. In this section, we show the hardware resource utilization in this study and compare the results with other previous works. In addition, it also compares this study’s implementation method with some previous works.

Since it is difficult to evaluate the detection rate on a FPGA, a simulation is conducted using software to estimate the accuracy degradation of the proposed system with the fixed-point implementation. The software with a fixed-point implementation has the same behavior as the proposed system with a fixed-point implementation. We also implement floating-point arithmetic in the software and compare it with its fixed-point implementation to estimate the accuracy degradation. Finally, we compared the fixed-point implementation of the software with Dalal’s implementation [10].

### 4.1. Implementation Method and Resource Utilization

Table 2 compares this study’s implementation method with other previous works and Table 3 compares this study’s resource utilization with other previous works. Compared to the implementation from Kadota et al. [16], which just concentrates on the HOG feature extraction without implementing the classification, this study implements not only the HOG feature extraction, but also the SVM classification. Although they do not use any memory in their platform, this study uses significantly less LUTs and registers. Compared to the implementation from Negi et al. [17], the memory utilization in this study is significantly lower than theirs, and the resolution is higher. In the implementation from Hsiao et al. [18], their LUTs, registers, DSP blocks, and memory are all less than this study’s. However, their implementation is not accomplished all in hardware. Their implementation is accomplished in an embedded hardware/software co-design. They just implement the HOG feature extraction in FPGA hardware and implement the SVM classification in an embedded ARM processor. Comparing this study’s resource utilization of HOG (without the SVM classification) with their resource utilization, the resource utilization between this study’s and theirs does not have significant difference, but the proposed implementation can achieve a higher frame rate. Compared to the implementation from Komorkiewicz et al. [19], their implementation used the single-precision floating point representation in all stages of image processing, so their resource utilization was very large. In the implementation from Hiromoto et al. [20], although they used fewer DSP blocks than the proposed system, the proposed system used significantly fewer LUTs and memory than theirs. Compared to the implementation from Mizuno et al. [21], theirs and this study use the same platforms, but the study does not use any external memory modules. Especially in the SVM classification module, they use an external SRAM to store SVM coefficients and intermediate results. In this case, the SRAM can help the FPGA to reduce its memory utilization significantly, but it does not indicate that they truly reduce the memory utilization. They just use the external memory (SRAM) instead of the internal memory (the memory of the FPGA).

### 4.2. Detection Rate

To evaluate the detection rate of this study, a simulation is conducted using software for object detection to estimate the performance and the accuracy degradation. The software has the same behavior as the FPGA implementation and it is implemented by using Microsoft Visual C++ 2013 Express Edition with the OpenCV library version 3.1 and two different databases.

**Figure 8:**
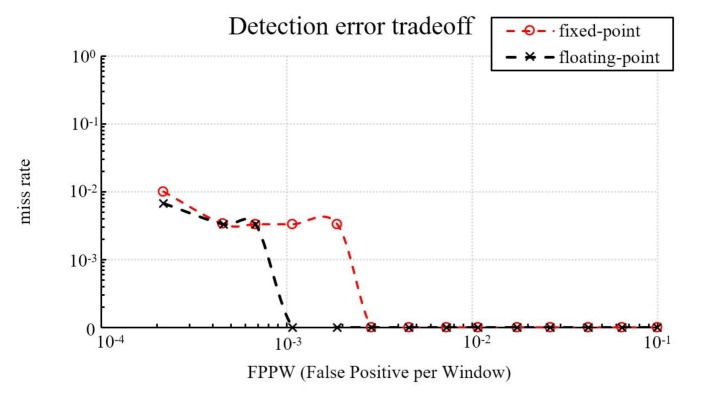
Detection accuracy of fixed-point and floating-point implementations on the MIT pedestrian database.

The first database used in this study is the well-established MIT pedestrian database [28]. This study selects 624 images with at least one person each as positive training examples and 300 images with at least one person each is selected as positive testing examples. In negative examples, since the MIT pedestrian database does not have person-free images, we selected 3120 patches sampled randomly from 312 person-free scenes in the INRIA person database [29] as negative training examples. Subsequently, we select another 1160 patches sampled randomly from 116 person-free scenes in the INRIA person database as negative testing examples. Figure 8 shows the detection accuracy of fixed-point and floating-point implementations in this study, which presents a graph of false positives per window (FPPW) versus the miss rate. The detection accuracy of both implementations in this study are less than, or equal to, 1% at any location on the FPPW axis.

**Figure 9:**
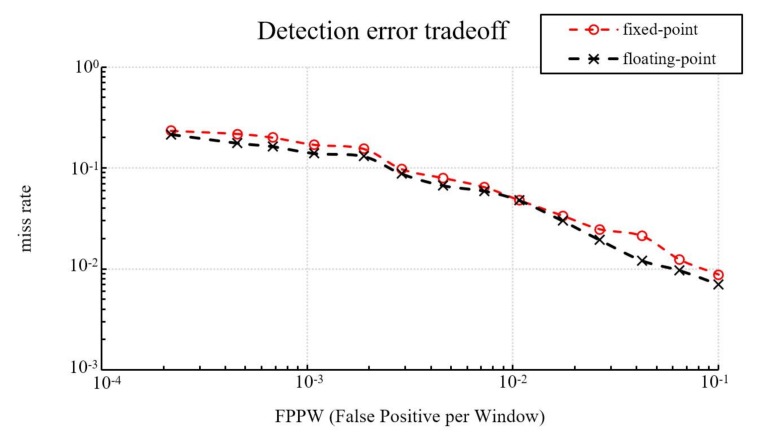
Detection accuracy of fixed-point and floating-point implementations on the INRIA person database.

The second database used in this study is the INRIA person database [28]. This study selects 1208 images with at least a person each as positive training examples, together with their left–right reflections (2416 images in all). A total of 12,180 patches sampled randomly from 1218 person-free scenes are selected as negative training examples. In the testing period, this study selects another 566 images as positive testing examples, together with their left–right reflections (1132 images in all), and this study selects another 4530 patches sampled randomly from 453 person-free scenes as negative testing examples. Figure 9 shows the detection accuracy of our fixed-point and floating-point implementations. The miss rate of the fixed-point is 3% higher than the floating-point at 10−3 FPPW, with the same miss rate at 10−2 FPPW, and 0.17% higher at 10−1 FPPW. On average, the fixed-point has a 1.42% higher miss rate than the floating-point.

There are several factors that would cause the differences in the miss rate when the fixed point parameters are used to replace the floating-point parameters, such as:

1. Quantization errors in the weights when converting *RGB* channels to the gray channel.
2. Quantization errors in the values of tan*θ* when computing the bin boundaries.
3. Truncation of the non-integer parts when taking square roots in the gradient magnitude calculation.
4. Truncation of the non-integer parts when taking square roots and divisions in the block normalization calculation.

**Figure 10:**
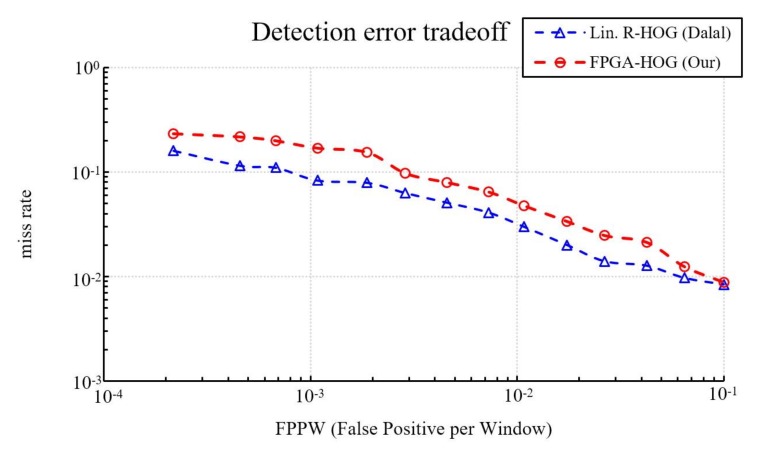
Comparing our fixed-point implementation with Dalal’s on the INRIA person database

In addition to evaluating the detection rate of fixed-point and floating-point implementations, this study also compares fixed-point implementations to Dalal and Triggs’s pure software implementation [10]. As shown in Figure 10 our miss rate is about 8.66% higher at 10^−3^ FPPW, 1.77% higher at 10^−2^ FPPW, and 0.04% higher at 10^−1^ FPPW. On average, the proposed hardware implementation has about a 4.05% higher miss rate than the original software-only HOG design. Other than the quantization errors described previously, the additional differences in the miss rate is caused by the omission of bilinear interpolation in bin voting.

## 5. Conclusions

This study implements the HOG pedestrian detection algorithm in FPGA, and by changing three sub-modules in the HOG flow, the proposed system can achieve a higher frame rate with only little degradation in accuracy. Through simplifications in the Getting Bin, it can use fewer hardware resources to determine the bins of the histogram. The Cell Histogram Generation sub-module exploits the distributed writing and two shift registers to easily deal with each intermediate cell histogram without any address decoder. In the Block Histogram Normalization sub-module, it reuses each sum of cell histogram, which is overlapped between each block to decrease the calculation workload. Moreover, to reduce the memory utilization, the SVM classification module in this study also uses a cell-based scanning structure. It separates a 3780-feature image into 105 steps of the partial SVM classification. When descriptors of a block are completed in the HOG module, it can begin the partial SVM classifications in the SVM classification module. Finally, rows of detection window positions, columns of detection window positions, and the results of detection windows would be outputted.

Compared to previous implementations, this study realizes both the HOG algorithm and the SVM classification on a FPGA without using any external memory modules to achieve a real-time pedestrian detection under a resolution of 800 × 600. Shown in experimental results, the proposed system’s resource utilization of the FPGA is lower than these implementations, relatively, and the average detection rate is slightly decreased.

## Figures and Tables

**Table 1:** Key performance parameters of the digital image sensor.

Parameter		Value
Active Pixels		2592 H × 1944 V
Pixel size		2.2 μm × 2.2 μm
Color filter array		RGB Bayer pattern
Shutter type		Global reset release (GRR)
Maximum data rate/master clock		96 Mp/s at 96 MHz
Frame rate	Full resolution	Programmable up to 15 fps
	VGA mode	Programmable up to 70 fps
ADC resolution		12-bit
Responsivity		1.4 V/lux-sec (550 nm)
Pixel dynamic range		70.1 dB
SNRMAX		38.1 dB
Supply Voltage	Power	3.3 V
	I/O	1.7 V–3.1 V

**Table 2:** Comparing this study’s implementation methods with other previous works.

	[16]	[17]	[18]	[19]	[20]	[21]	Proposed
Implementaion Device	HOG-FPGA SVM-No	HOG-FPGA AdaBoost-FPGA	HOG-FPGA SVM-ARM Processor	HOG-FPGA SVM-FPGA	HOG-FPGA SVM-FPGA	HOG-FPGA SVM-FPGA	HOG-FPGA SVM-FPGA
External memory	×	×	ARM processor	×	×	SRAM	×
Distributed writing and two shift registers in Cell Histogram Generation	×	×	×	×	×	×	○
105 blocks of the SVM classification	×	AdaBoost	×	×	×	○	○

**Table 3:** Comparing this study’s resource utilization with other previous works.

Platform	[16]	[17]	[18]	[19]	[20]	[21]	Proposed
	Altera Stratix II	Xilinx Virtex-5	Xilinx Spartan-6	Xilinx Virtex-6	Xilinx Virtex-5	Altera Cyclone IV	Altera Cyclone IV
Resolution	640 × 480	320 × 240	various ^1^	640 × 480	320 × 240	800 × 600	800 × 600
Frame rate (fps)	30	62	about 15 ^1^	60	38	72	162
Operating frequency (MHz)	127	44	192	25	167	40	150
# of LUTs	37,940	17,383	4169	113,359	28,495	34,403	16,060
# of registers	66,990	2181	3533	75,071	5980	23,247	7220
# of DSP blocks	120	no data	10	72	2	68	69
Memory (kBit)	no data	1327	243	4284	2196	348	334

^1^ In the implementation of Hsiao et al., their inputted images are from four datasets, which have various size of images. They just say their implementation can reach about 15 fps.
